# Patient Satisfaction Measurement: A Comparison of Likert and Item-Specific Response Options Scales

**DOI:** 10.3390/healthcare13233017

**Published:** 2025-11-21

**Authors:** Vassilis Aletras, Stavros Chatzopoulos, Maria Kalouda, Dimitris Niakas, Angeliki Flokou

**Affiliations:** 1 Department of Business Administration, School of Business Administration, University of Macedonia, 54636 Thessaloniki, Greece; 2 School of Social Sciences, Hellenic Open University, 26335 Patras, Greece; std161862@ac.eap.gr (M.K.); dniakas@med.uoa.gr (D.N.); 3 Department of Statistics, University of Western Macedonia, 50150 Kozani, Greece; schatzopoulos@uowm.gr; 4 Medical School, National and Kapodistrian University of Athens, 11527 Athens, Greece

**Keywords:** patient satisfaction, psychometric evaluation, questionnaire design, Likert scale, Item-Specific Response Options (ISRO), confirmatory factor analysis (CFA), Cronbach’s alpha

## Abstract

**Background/Objectives**: Patients’ reports on their satisfaction with the care received often have been seen as a key quality indicator of hospital performance. However, the potential effect of different approaches to its measurement has not been adequately assessed in the health care setting. This study therefore aimed to methodologically compare two different response formats in patient satisfaction questionnaires—Likert scales and Item-Specific Response Options (ISRO)—within a Greek public hospital context. The aim was to comparatively explore resulting item- and scale-level score values, ceiling effects, acquiescence bias, and psychometric properties, including reliability and validity. **Methods**: An overall sample of 400 hospitalized patients at a National Health Service general university hospital was randomly assigned to two groups during February–March 2025. One group completed a Likert-scale questionnaire and the other a questionnaire, with the same content, that employed an ISRO format instead. The questionnaire items covered two aspects of the hospital experience, these being the satisfaction with doctors/nurses as well as the organization and planning of care. Statistical analysis involved Kolmogorov–Smirnov tests for normality, descriptive statistics, chi-square and Fisher’s exact test, *t*-tests, Mann–Whitney tests, ceiling effects, regressions, Cronbach’s alpha coefficients, and confirmatory factor analysis (CFA), with measures of composite reliability and average variance extracted and model fit indices. **Results**: Our analysis identified differences in the distributions of patient responses for many items, including variations in median values and the proportion of positive answers. ISRO items tended to produce higher ratings for nursing care and overall satisfaction, whereas Likert items yielded higher scores in organizational aspects. However, the magnitude of these differences was generally small. Regression analysis, adjusting for length of stay, confirmed statistically significant but modest differences in scale scores between formats. Neither format was superior in terms of ceiling effects, whereas no consistent evidence of acquiescence bias was found. Psychometric testing showed that Likert scales had somewhat higher internal consistency reliability and convergent validity, while ISRO exhibited a better model fit in CFA. **Conclusions**: The item response format seems to affect reported satisfaction scores, yet the impact is rather limited in practical terms for decision-making. Since neither format is consistently superior, the choice between them should depend on study aims, respondent burden, and the intended use of satisfaction scores by policy makers. Moreover, concerns about acquiescence bias may have been overstated in the health care context. Future research should extend these comparisons with other instruments and larger and more diverse samples, as well as employ complementary methods to clarify how response format affects patient satisfaction measurement.

## 1. Introduction

According to the World Health Organization, health care systems across the globe are expected to achieve multiple, interrelated goals. Not only should they pursue efficiency, equity, and improvements in population health, but they must also enhance responsiveness. The latter involves the legitimate expectations of the population for non-health-enhancing dimensions of their interactions with the health system, like dignity, autonomy, confidentiality, prompt attention, amenities, and quality of patient–provider interaction. Being responsive and providing people-centered care is essential since it relates to basic human rights and improves information flow in provider–patient encounters [1,2]. Furthermore, some studies [3] have suggested that higher patient satisfaction might lead to better health outcomes in some contexts, whereas others [4] have suggested that patient experience and clinical outcomes are likely to represent distinct aspects of quality of care, and the former should be seen as a marker of patient-centered care and good customer service, rather than a proxy for clinical outcomes. Patient experience and satisfaction also have financial impacts. Satisfied patients are more likely to return to the hospital and might even refer friends by word of mouth. Thus, unsurprisingly, health care systems often monitor their hospitals with patient experience or satisfaction surveys that they report publicly in order to foster transparency, accountability, performance assessment, and improvement. Tentative examples of such reporting can be found in countries such as the UK [5] and Australia [6].

Patient satisfaction and patient experience can both be elicited via surveys and yet are different concepts. Patient satisfaction with health care is considered an outcome measure of patients’ experience with care and reflects their ultimate judgment, from a legal and rights perspective [7]. It is, in fact, formed from the experiences patients have from the actual encounter with a health care provider and their needs, expectations, and values, which are in turn affected by their characteristics (e.g., health status, age, educational level, past contacts). The patient assesses and states whether or not the care provided has met their needs and expectations regarding various aspects of care (e.g., staff behavior, staff timeliness, food, cleanliness, adequacy of information) in questionnaires designed by researchers. A patient’s experience of care provided may have a direct influence on satisfaction, as well as an indirect effect by affecting their needs, expectations, and values, which in turn affect satisfaction. Expectations are, in turn, further shaped by broader societal, community, and family contexts [8]. In the same framework of patient-centered care, patient experience is a process indicator that captures the interpersonal aspects of the quality of care received and involves effective communication, respect and dignity, and emotional support [9].

Patient experience measures can be used for quality improvement interventions and monitoring [8]. Since they are more objective and are sensitive to differences in quality care across different providers, institutions, or time, they can be used to identify performance gaps or evaluate quality changes after the initiation of interventions or policies. On the other hand, patient satisfaction and patient experience survey data can be employed to increase health system accountability [8]. The survey results on satisfaction, however, should be approached carefully to better understand and account for inherent subjectivity during the interpretation of ratings. This is especially true when making comparisons across institutions since the impact of case-mix and patient demographics may play a substantial role that may even require appropriate correction. Qualitative research should complement quantitative patient satisfaction surveys.

Patient satisfaction measurement with the use of questionnaires has been widely researched in the literature for hospital care [10,11,12]. Many of the studies explored the predictors of patient satisfaction. Hospital survey data in Norway showed that both fulfillment of expectations and patient-reported experiences are the most important predictors of overall patient satisfaction with hospital care. This implies that subjective expectations are indeed important in shaping satisfaction or dissatisfaction in questionnaire reporting [13]. Nevertheless, experiences with aspects of care are also important in determining overall satisfaction. For instance, a Chinese study has found experience with nursing care to be the most important predictor of overall satisfaction [14]. Finally, other factors related to satisfaction, namely structural (e.g., length of stay, nurse staffing, nursing hours per patient day, cleanliness, waiting times), process (e.g., patient perceived nursing care, nursing care quality, communication with nurses and doctors, treated with courtesy and respect, being listened), and demographic characteristics (e.g., gender, age, education level, mental status, self-perceived health status, income) have been found to affect patient satisfaction, with the statistical significance and direction of the effects not always being consistent across studies [15,16,17,18].

The above-mentioned research has been conducted using different questionnaires with different response scales and even lengths of these scales. However, an effective questionnaire for measuring patient satisfaction should represent patients’ actual views of their hospital care, not artifacts of measurement error or bias. It should therefore be reliable and valid. Validity refers to the extent to which an instrument measures what it is intended to measure, encompassing concepts such as content validity, construct validity, criterion validity, and known-groups validity. Reliability refers to the degree to which an instrument consistently measures a construct, producing stable and repeatable results, and is typically assessed through internal consistency reliability, test–retest reliability, and inter-rater reliability [19,20,21,22].

The Likert scale is perhaps the most widely used response format in the literature of patient satisfaction. It is, in principle, an ordinal scale in which respondents are presented with various statements and are asked to state their level of agreement or disagreement using scales that vary in length across studies. Although ordinal in nature, it has been argued that researchers can use typical descriptive statistics and tests rather than merely reporting frequencies [23,24]. It has also been found that ordinal methods may give very similar findings to parametric methods regarding satisfaction constructs in home care [25].

One of the most important advantages of the Likert (agree/disagree) scale is its simplicity and ease of use. There is only a need to present the agreement scale once, at the start of a battery of questions. Hence, it may be easy for respondents to understand and complete with little cognitive burden and efficient for researchers to administer and analyze [26]. It is also widely used in practice, therefore allowing comparability across studies. On the other hand, Likert scales are symmetrical and might be prone to central tendency bias; that is, respondents may avoid extreme categories and clustering around the middle might be observed [27]. Moreover, acquiescence bias might be present in that the respondents might exhibit a tendency to agree with the statement described in an item, regardless of the content [28]. Another issue raised is the potential mismatch, for some items, between the statements presented and the response options (i.e., the agree/disagree response options are not appropriate for replying to the statement as framed), making it, in fact, difficult for patients to process [29].

An alternative that has been proposed to overcome such problems is the Item-Specific Response Options (hereafter referred to as the ISRO) scale. This presents individuals with a direct question (rather than a statement to which they are asked to agree or disagree) and a set of tailored response categories that match its content (e.g., frequency, quality, extent). For instance, the Likert statement “The nurse explained things clearly” with response options from “Strongly agree” to “Strongly disagree” is framed as “How often did the nurse explain things in a way you could understand?” with response categories now being “Never”, “Rarely”, “Sometimes”, “Usually”, “Always”. A number of studies have thus examined whether the ISRO outperforms Likert in terms of construct validity, acquiescence bias, content validity, quality of data, and reliability [29,30,31,32]. One should, however, not overlook the potential drawbacks that might be associated with ISRO scales, namely the greater cognitive effort and time required to complete them, since they do not homogeneously present all items [30], and reduced comparability across items, since response options vary by question [33]. They also entail greater design complexity, requiring greater research effort during questionnaire design.

Due to the limited number of available studies and the fact that almost all data employed come outside the health care sector, the aim of the present research was to compare Likert-type and item-specific (ISRO) scales in the assessment of patient satisfaction, by examining their item- and scale-level scores, evaluating their psychometric properties, and investigating the presence of acquiescence bias as a potential source of measurement error. To achieve this, we randomized the administration of the two questionnaires to two samples of patients who were admitted to a Greek public hospital.

## 2. Materials and Methods

### 2.1. Aim and Study Protocol

The present research aims to compare two questionnaires for the measurement of patient satisfaction with hospital care. Specifically, it measures self-reported patient satisfaction with medical and nursing care, as well as with the organization and planning of care, and also assesses the psychometric properties and acquiescence bias of the two instruments. Factors that might affect self-reported patient satisfaction are also examined. The two questionnaires under study include items with equivalent content, measuring the same aspects of patient satisfaction. One, however, uses the Likert scale, whereas the other uses an Item-Specific Response Options scale. Each of the instruments was completed by 200 participants aged 18 and above hospitalized in the University Hospital of Larissa, in the period 7 February 2025–16 March 2025. The study was approved by the Academic Program Committee of the Postgraduate Program in Health Care Management (DMY) at the Hellenic Open University (20 January 2025) and by the Scientific Committee of the University General Hospital of Larissa, Greece (Protocol No. 5650; 6 February 2025). Written consent was acquired from all participants prior to questionnaire completion.

### 2.2. Study Instruments

The questionnaire on which we based our survey consists of 18 items. These partly comprised a 33-item instrument previously developed by Aletras et al. (2009) [34] through literature review, interviews with health professionals, and on-site observation, as well as a pretesting phase involving concurrent and retrospective think-aloud cognitive interviews with patients to ensure item clarity and content validity. An exploratory factor analysis in that study resulted in four scales. To safeguard respondents’ willingness to complete the survey in the present research, two of these scales were selected and designed in two alternative versions. These measure self-reported patient satisfaction with doctors and nurses as well as with the organization and planning of care in the hospital. We also included 1 item measuring the overall experience/satisfaction with the care provided in the hospital. The first scale, entitled “Contact with doctors and nurses”, includes the following 12 items capturing patients’ satisfaction: interest/care by nurses, politeness/rudeness of nurses, information by nurses regarding medical procedures, nurses’ willingness to answer patients’ questions, discussion of patients’ concerns and fears with nurses, timely or delayed response of nurses to patient calls, nurses’ respect for patients’ dignity, nurses’ discretion, frequency of physicians’ visits for patient health checks, information by physicians regarding patients’ health status, physicians’ discretion towards patients, physician availability, and willingness to respond to patients’ questions. The second scale, namely “Organization and planning of care”, involved six items in the original work: waiting for an available bed, pleasant/unpleasant hospital admission process, timely/delayed completion of procedures, organization of patient stay and avoidance of errors, discharge before patient feels ready for safe transition, and timely discharge from hospital on the day of departure. The last item was omitted here since, at the time patients were presented with the questionnaire, the discharge process was not completed yet and therefore patients could not assess it and express their scores. Thus, our second scale consists of 5 items.

The scales outlined above were shown in Aletras et al.’s (2009) study [34] to have satisfactory psychometric properties. Specifically, their Cronbach’s alpha was 0.957 and 0.851, therefore indicating internal consistency reliability. Moreover, intraclass correlation coefficients, used as measures of test–retest reliability, were also very high, taking the values 0.978 and 0.971. Since it was a telephone survey, interrater reliability was also assessed, with the correlation coefficients being 0.970 and 0.938. The multitrait–multimethod approach, which compares correlations of items with their own scale with those of the same items with other scales, showed very good construct validity. Validity was also corroborated by high Kendall’s Tau correlations of reported waiting times with patients’ satisfaction regarding waiting. Finally, the Spearman correlations of reported overall patient satisfaction, as measured by a single item, with the two scale scores were 0.567 and 0.652.

Since our aim in the present study was to comparatively evaluate different scales and the questionnaire previously developed was expressed on a 5-point Likert scale (Strongly agree to Strongly disagree), we converted the items—Likert statements—into 5-point Item-Specific Response Options formatted questions. Despite an apparent debate among researchers, this length of options has been found to have adequate precision [35,36,37]. In addition to its documented reliability and validity, this 5-point scale length was preferred because it was used in our original 2009 study; it is a very popular length in the applied literature on patient satisfaction, and it is easy to use and involves little cognitive burden on fragile patients ready to leave the hospital.

Conversions from Likert to ISRO were meant to keep the content of the items unchanged. For instance, the negatively worded Likert item “Nurses were rude” (Strongly agree, Agree, Neither agree nor disagree, Disagree, Strongly Disagree) was expressed on the second questionnaire as an ISRO question: “Which of the following better characterizes the way nursing staff treated you? Would you say it was… Very polite, Somewhat polite, Neither polite, nor rude, Rather rude, Very rude”. Another example is the positively worded item “The nurses showed interest in me during my hospitalization” (Strongly agree… Strongly disagree), which was converted into “What was the attitude of the nurses towards you during your hospitalization? Would you say they showed: Great interest, Moderate interest, (Neutral) Neither interest nor indifference, Somewhat indifferent, Completely indifferent”.

Pretesting involved 40 patients. The aim was twofold. First, 10 individuals were interviewed with the think-aloud method to ensure that Likert and item-specific (ISRO) versions of an item were semantically equivalent before comparing the formats in the main study. This process showed that there were no comprehension or content issues that could obscure study findings. Moreover, 15 patients were asked to complete the Likert format and another 15 the ISRO, while the time of completion was monitored by the research team. Completion time was about 6 min for the Likert and 7 for the ISRO. Although this might imply that the cognitive burden on patients of the ISRO is higher, our measurement only comes from a few patients and can only be considered indicative. We did not measure completion time in the subsequent main study since we wanted patients to privately and undistractedly complete the questionnaire and put it in a ballot box to preserve anonymity.

Demographic questions involved gender (male, female), age (18–30, 31–44, 45–60, 61–74, 75+), education level (elementary or lower, secondary, higher), household’s financial condition (we are unable to make ends meet; we manage, but with great difficulty; we manage, but have little left over; we are financially comfortable; don’t know/don’t want to answer), and self-reported health status (very good; good; fair; poor; very poor). Finally, we included length of stay since it has been found in some studies of overall satisfaction or certain aspects of it that it affects negatively stated values in at least some dimensions of satisfaction [38,39].

### 2.3. Questionnaire Administration and Sample

A sample of 400 patients hospitalized during the period 7 February–16 March 2025 was obtained. Eligibility criteria involved participants’ age (≥18) and adequate knowledge of the Greek language. Exclusion criteria included hospitalization in the intensive care unit, not being able to hear or speak, and mental or cognitive disorders.

Convenience sampling was employed to collect the required data for analysis. This method was selected taking into account practical aspects, aiming to include as many patients as possible, from almost all the clinics of the University Hospital of Larissa. Patients were approached until the required sample size was reached. Twenty-eight individuals refused to participate in the survey (response rate 93.46%). In total, 200 participants completed the Likert questionnaire and 200 answered the respective ISRO. The two instruments (Likert, ISRO) were administered to eligible patients using a self-administered format during their hospital stay, while awaiting their discharge. Participants were, in fact, randomized to receive one of the two questionnaires. It was handed to them along with a letter informing them about the research (purpose, process, participants’ anonymity) and asking for their consent. The ballot box method was employed to collect the self-administered questionnaires. That is, patients completed the questionnaires privately, without interviewer assistance, and instead of handing it back to staff or the researchers, they placed it into a sealed box to which only the research team had access. This method was chosen to ensure anonymity, reduce social desirability bias, and encourage honest responses.

### 2.4. Statistical Analysis

Negatively worded items were re-coded in the completed Likert-type questionnaires in order for increasing scores to indicate better experience (higher satisfaction). Qualitative and categorical variables are presented as absolute and relative frequencies. For continuous variables and interval/ordinal scale data, descriptive statistics involve means, standard deviations, medians, interquartile ranges, and min and max values. Kolmogorov–Smirnov tests were also employed to assess normality. Since, often, in patient satisfaction studies, negative skewness of the scores’ distribution results in high ceiling effects for questionnaire items, we additionally computed the percentage of observations taking the maximum value, 5, for each item.

χ^2^ tests for independence were used for exploring associations between categorical variables, along with Fisher’s exact tests, where appropriate. Relationships between continuous or interval/ordinal variables were assessed with *t*-tests for independent samples or non-parametric Mann–Whitney tests. Regression analysis was employed to disentangle the effect of questionnaire type from the impact of length of stay on scale scores. In addition, we initially examined the possibility of treating the latter variable as a variable for evaluating known-groups validity. The rationale for our consideration was that extended stays typically reflect more complex cases, greater exposure to organizational or care-related challenges, and increased patient fatigue, which may reduce overall satisfaction. However, empirical evidence is in fact mixed regarding the statistical significance and the directionality of this relationship [39,40,41,42]. Hence, length of stay was not used for evaluating known-groups validity in the present study.

Confirmatory factor analysis was conducted with Maximum Likelihood estimation with Bootstrap correction in order to compare the structural validity of the two questionnaires [43]. The following goodness-of-fit indices were employed: Root Mean Square Error of Approximation (RMSEA), Goodness-of-Fit (GFI), Comparative Fit Index (CFI), Tucker–Lewis Index (TLI), and Average Variance Extracted (AVE). The minimum acceptable levels based on the literature are also reported. Specifically, a model is acceptable if RMSEA is less than 0.08, CFI/TLI/GFI is greater than 0.90, χ^2^/df is less than 3, and factor loadings exceed 0.50 [44,45]. Moreover, internal consistency reliability was measured with Cronbach’s alpha coefficient (acceptable if greater than 0.70). The literature also suggests that, in general, Composite Reliability and Average Variance Extracted should be above 0.60 and 0.50, respectively [46]. Finally, we did not assess known-groups validity by examining whether relationships, previously established in prior research, between patient satisfaction scales and demographics are validated in one or both the Likert and ISRO samples. This is because, as mentioned in the Introduction, the relationships seem to lack consistency regarding statistical significance or direction. Statistical significance was set at 5%. SPSS v. 23 (IBM Corp., Armonk, NY, USA) and JASP v. 0.18.3 (Department of Psychological Methods, University of Amsterdam, Netherlands) were used to analyze the data.

## 3. Results

The characteristics of the two samples (Likert, ISRO) are shown in Table 1. Appropriate statistical tests, according to the normality test results, were conducted to assess whether differences exist between participants’ characteristics in the two groups.

Upon inspection, it becomes evident that most study participants are female, in the age groups 45–60 and 61–74. Most had a tertiary education, and more than 85% considered their health as good or very good at the time of the survey administration. Furthermore, the great majority of households in the two samples stated that they coped with their financial obligations, but with little income left over. The average patient stayed in the hospital for approximately 5 days. Randomization has generally been successful. Differences are insignificant in all participant characteristics except length of hospital stay. The impact of this variable should be further examined since, in some studies, it has been found to affect patient-reported satisfaction.

Normality tests for questionnaire items indicated that the normality assumption does not hold (*p* < 0.001). Table 2 reports the descriptive statistics of the items for the two questionnaires (Likert and ISRO), together with the results of Mann–Whitney tests for potential differences in their distributions. We notice that for 10 out of 18 items, there are statistically significant differences. Taking into account that the items under comparison are expressed on five-point scales, we see that the means are quite similar in some cases, with the difference in the values in the Likert and ISRO not exceeding 0.15 (items “politeness of nursing staff”, “respect shown by nursing staff”, “well-organized hospital stay without errors”). For some other items (“promptness of nursing staff”, “overall satisfaction”, “monitoring of patients’ health by doctors”), the differences in means are somehow higher, that is between 0.20 and 0.35, and yet for other items, they are even higher and with more practical significance (“clarity of instructions of nursing staff for procedures”, “waiting time for a bed”), in the approximate range of 0.70 to 1.20.

Furthermore, in the five items measuring the satisfaction with the nursing staff and having significant differences in the two questionnaires, ISRO formatting yielded higher mean values than Likert. Similarly, the overall satisfaction of the average patient was also higher in that questionnaire. In contrast, Likert formatting resulted in a higher average score only for one item regarding doctors, with all other “doctor” scores being similar. The Likert questionnaire also had higher mean values compared to ISRO in three out of five items referring to the organization and planning of care within the hospital, with the other two items lacking statistical significance. The picture is thus mixed. It should also be noted that these differences cannot be attributed to the positive or negative wording of the Likert items, since in many cases, a positive Likert item had a lower mean value than its respective ISRO, and vice versa.

Moreover, we should consider the fact that the items have not been found to be normally distributed. The median is therefore generally considered a more appropriate measure of central tendency. This differs in four items by a notable size of one point. In three of them (“waiting time for a bed”, “timeliness of scheduled procedures”, “monitoring of patients’ health by doctors”), the Likert questionnaire yielded higher values, whereas in one (“clarity of instructions of nursing staff for procedures”), it produced a lower value. The dispersion of participants’ answers to the questionnaires differed only in five items, with four of them having a higher interquartile value in the Likert format and one in the ISRO. Finally, the picture is mixed regarding the ceiling effects of individual items, with some exhibiting higher effects in Likert and others in ISRO.

Since means and medians collapse all response categories into one summary number, they might conceal potential differences in respondents’ use of the upper response categories, in which acquiescence tends to show. Hence, to assess acquiescence bias more directly, in Table 3, we compare the percentage of respondents selecting the top two response categories (i.e., those reflecting a positive view of a hospital service) in the Likert and ISRO questionnaires. In other words, we examine if more individuals select “agree/strongly agree” in positively worded items (or “disagree/strongly disagree in negatively worded ones) in Likert than select the equivalent top categories (e.g., “somewhat polite/very polite”) in ISRO. On inspection, it is apparent that in 8 out of 18 items, there are statistically significant differences. In three items, the Likert percentage of positive responses is higher, whilst in five, the ISRO prevails. Therefore, again, there is no evidence to suggest, in general, that Likert is more susceptible to acquiescence bias than ISRO.

The descriptive statistics of the two scale scores are shown in Table 4. The distributions of scale scores of satisfaction are significantly different in the two samples. In the scale regarding the contact with doctors and nurses, the mean and median are lower in the Likert questionnaire. The opposite is apparent for the organization and planning of care scale. The dispersion of respondents’ answers was greater for Likert in both scale scores. Ceiling effects are similarly low in both questionnaires, with no format being superior.

Two regression equations were run with Ordinary Least Squares estimation. Each had one of the two scales (Contact with doctors and nurses, Organization and planning of care) as the dependent variable. A dummy variable for the type of questionnaire that was used to collect the data (Likert, ISRO) and the patient’s hospital length of stay constituted the regressors. Since the length of stay might affect patient-stated satisfaction and was found to differ in the two sub-samples (see Table 1), we corrected for this effect in order not to confound differences in patient satisfaction scores evidenced between the Likert and ISRO questionnaires. As seen in Table 5, length of stay has a negative impact on satisfaction, albeit marginally insignificant in one equation. This suggests that increased stay at the hospital is associated, on average, with a lower level of satisfaction. Most importantly, after adjusting for this influence, the Likert questionnaire is associated with lower patient satisfaction (by approximately 0.2 units, on average) with the contact with doctors and nurses and higher satisfaction (by 0.23 units) with the organization and planning of care compared to the ISRO one.

We also conducted a sensitivity analysis to further assess the effect of length of stay on study results. Specifically, we applied the two regression models for length of stay ≤2 days, ≤3 days (i.e., the 1st quartile of length of stay for both questionnaires), for length of stay ≤5 days and >5 days (that is, a value approximately equal to the mean and median of length of stay), for length of stay >6 days (3rd quartile of length of stay), and for length of stay >7 days. Table 6 reports regression results following patient stratification. It is apparent that the pattern of associations between response format and satisfaction scores remained largely consistent with our initial analysis. That is, the effect of length of stay was once more smaller and, in general, not statistically significant in the stratified samples. Therefore, it seems that our initial conclusions are robust to observed length of stay differences.

In order to assess structural validity, we present in Table 7 the goodness of fit indices of the confirmatory factor analyses applied to the data from the two questionnaires. The model for the Likert format does not have a satisfactory fit since none of its indices (RMSEA, χ^2^/df, GFI, CFI, TLI) takes acceptable values within the acceptable ranges. Specifically, RMSEA and χ^2^/df have unacceptably high values (0.126 and 4.152) and CFI and TLI fall well below the 0.90 threshold. In contrast, the ISRO model exhibits adequate fit, having χ^2^/df and RMSEA within the acceptable ranges (i.e., 1.988 ≤ 3 and 0.071 ≤ 0.08, respectively) and the GFI index (0.869) marginally close to the desired threshold level (≥0.90). Although CFI and TLI values do not reach the cut-offs of 0.90, they remain higher compared to the Likert model, suggesting better overall fit. Therefore, the findings of CFA imply that ISRO is slightly better in terms of structural validity, although it still provides weak support for the two-factor structure.

In the Likert questionnaire, the Spearman correlation between the two scales (“Contact with doctors and nurses” and “Organization and planning of care”) is moderate (rho = 0.499). The fact that it is not very high might imply an adequate discriminant validity in the sense that the two dimensions are distinct enough to be considered separate constructs and do not measure the same aspect of patient satisfaction. Moreover, the fact that the correlation is not very low suggests convergent validity, in that the two scales might be parts of the same construct, namely patient satisfaction with care. On the other hand, in the ISRO instrument, the respective correlation is substantially lower (rho = 0.234), corroborating discriminant validity but perhaps raising doubts about convergent validity. Additional evidence in favor of the higher convergent validity of the Likert format comes from the Spearman correlations between the two scales and the item measuring overall satisfaction, being 0.392 (Contact with doctors and nurses) and 0.527 (Organization and planning of care) for the Likert design and 0.358 and 0.277 for the ISRO, respectively.

In Table 8, the factor loadings and psychometric measures of the Confirmatory Factor Analysis for the two questionnaires are presented. It is apparent that for most items, Likert displays higher factor loading values than ISRO. Internal consistency reliability—as measured by Cronbach’s alpha—is higher in the Likert format. Interestingly, by dropping two items from the two scales, its values are improved to 0.723 and 0.705. In contrast, alpha cannot be improved by deleting items in any of the scales in the ISRO. Similarly, Likert outperforms ISRO by having higher values of composite reliability and average variance extracted.

## 4. Discussion

This study assessed item and scale scores resulting from the administration of two questionnaires meant to have the same content but different response options (Likert vs. Item-Specific). To ensure that patient-related characteristics would not confound the findings, randomization of these two printed versions to two groups of patients was employed, which was generally successful since there were no statistically significant differences between them regarding gender, age, level of education, household’s financial condition, and self-reported health status (Table 1). The only significant difference in the two groups refers to patients’ length of stay in the hospital, which was about 0.80 days between the two samples.

A point that needs to be further clarified is the choice of the between-subjects design in the present study. Alternatively, the within-subjects approach would have required the same individuals to consecutively answer both questionnaire formats. The latter could, in principle, offer stronger control for inter-person differences since it precludes unobserved confounding factors that may differ between subjects and can contaminate the observed differences between response formats.

In our research, the choice was guided by the following factors. Firstly, patients were approached inside the hospital prior to discharge. They were likely to be stressed, anxious, tired, and preoccupied with instructions and hence more prone to satisficing and careless completion of the questionnaire. In this context they might not be willing to answer many questions and the hospital administrators asked the research team not to put too much burden on them. Each response format involves 18 items (in addition to standard demographic and related questions). Therefore, doubling the burden for each patient was not an appealing option. Secondly, in line with the previous argument, we sought to avoid order, learning, or consistency effects that may threaten internal validity. Specifically, it might be the case that the order of the items might affect responses (e.g., patients might be tired, bored, or impatient and therefore complete less carefully over time). The accuracy and speed of their answers might also improve once they are familiar with the first format they are exposed to, and they might even try to show self-consistency by adjusting their later responses to earlier ones. Thirdly, reversing the order of response formats across sub-samples cannot fully remove all unwanted effects if the latter are in fact different depending on the order of testing. That is, in the presence of asymmetric response transfer, which is likely due to the different cognitive processing demands of Likert and ISRO scales, counterbalancing the order of response formats will not fully eliminate the effects, and the resulting contamination might persist [47]. Finally, the potential for confounding that is inherent in between-subjects designs has been addressed here by means of randomization and length-of-stay regressions.

Analysis of the Likert and ISRO questionnaires showed divergence in many mean item scores, though the magnitude varied, from negligible to differences up to 1.20 units (on a five-point scale). Overall, ISRO formatting tended to yield higher mean values for nursing staff-related items and overall satisfaction, while the Likert design showed higher average scores in some items concerning organizational and planning aspects of care and one doctor-related question, resulting in a mixed pattern. Moreover, median values differed by a full point in four items, most being higher in the Likert format, with the opposite only holding for clarity of instructions. Differences in the spread of patients’ opinions were also evident, yet only for five items, with Likert generally showing greater dispersion in the scores. Finally, no questionnaire was universally superior regarding ceiling effects.

Differences persisted also in the scale scores for “Contact with doctors and nurses” and “Organization and planning of care”. The difference in means between the Likert and ISRO scores was −0.21 and 0.28 in the two scales, respectively. For the medians, the respective figures were −0.17 and 0.25. That is, Likert took lower values for the first scale and higher for the second one. The higher values did not seem to result from the positive or negative wording in the agree/disagree scale since the second scale had proportionally more negatively worded items than the first scale and still had a higher mean value than ISRO. Furthermore, Likert also had a somewhat larger spread in the stated levels of satisfaction and the ceiling effects were small and similar for the two response formats. Together with the item-level findings, the evidence does not support the superiority of one of the two questionnaires in terms of sensitivity. It should also be noted that, in line with some previous studies [38,39], we found a negative effect of length of stay on satisfaction, as measured here by the two scale scores. We did not, however, treat this as a finding of known-groups validity since other studies have found a negative association in some instances (e.g., for certain specialties or certain questionnaire items) and a lack of statistical significance [40] or even a positive relationship in others [42].

In any case, there is evidence here that adjusting for this effect still results in statistically significant differences in patient-reported scores between Likert and ISRO, which were equal to −0.197 and 0.231 points in the two multi-item scales (Table 5). This conclusion is, in fact, robust to sensitivity analysis. Thus, a higher average value is evidenced for ISRO in the “Contact with doctors and nurses” scale and for Likert in the respective “Organization and planning of care” scale. Interestingly, although statistically detectable, the observed differences in scale scores (on a five-point scale) are rather small in magnitude and unlikely to be of substantive practical importance in most health care decision-making contexts. Nevertheless, they highlight the potential for response format to influence scores in ways that could matter in comparative reporting. That is, for decision-makers, these differences are usually considered too small to be practically important, unless results are used rather strictly for rankings, benchmarking, or accountability, where even small shifts may change outcomes.

Furthermore, the descriptive statistics in this study do not support the existence of a meaningful acquiescence effect in our sample. However, it could also be the case that the bias is small and cancels out when categories are averaged. Thus, the proportions in the top categories between Likert and ISRO for individual items were explored and showed mixed results that do not corroborate the hypothesis that the agree/disagree questionnaires are more prone to acquiescence bias. Similarly, Hanson (2015) [48] developed alternative item-specific response scales from a four-point agreement scale from the Crime Survey for England and Wales that measures attitudes towards the police. The hypothesis that the proportion of participants who agree with the statements in the Likert items is greater than the corresponding proportion of respondents who choose the top two response categories in the item-specific format was not confirmed, thus indicating the absence of acquiescence bias.

Their representative randomized experiment did not find evidence to support the hypothesis that, due to acquiescence bias, a greater proportion of respondents would agree with the statements in the agreement-scale questions than provide the equivalent responses (that is top two response categories) in the item-specific questions.

Confirmatory factor analysis that tested the structure found by the earlier work of Aletras et al. (2009) [34] resulted in statistics that indicate a better fit for the ISRO design. Specifically, only in that model is the RMSEA below the commonly cited threshold value of 0.08 [49]. This is despite the fact that the original 2009 questionnaire was, in fact, designed on a Likert scale.

The weaker CFA fit indices observed for the Likert version may partly reflect the methodological background of the instrument used, which was the only available validated questionnaire for measuring patient satisfaction in the Greek language. This was originally developed and validated via exploratory rather than confirmatory factor analysis. The present study therefore represents a more stringent assessment of its factorial validity and may have revealed structural ambiguities. A further possibility would be that the Likert format yields more homogeneous responses than ISRO (e.g., due to central tendency or acquiescence biases), which would make it harder for the hypothesized model (e.g., the two-factor structure) to fit the data well. However, the relatively poor fit does not seem to be caused by reduced variability in item responses since the standard deviations and interquartile ranges are higher for the Likert scales than for ISRO. Acquiescence bias was also not documented in our sample, as noted before. Since, in addition, the fit indices for both formats were not very satisfactory, we conclude that the earlier psychometric measure might not have performed very well on structural grounds.

Internal consistency reliability refers to the degree to which the items of a scale are correlated with each other and hence measure the same underlying construct. Cronbach’s alpha was higher in the Likert design and even achieved values above 0.70 in both scales by deleting one item in “Organization and planning of care”. Values in the range 0.70–0.95 are generally taken to imply sufficient inter-relatedness between items or homogeneous constructs and no item redundancy [50].

We also investigated the construct validity of the measurement models by assessing convergent and discriminant validity. Convergent validity is calculated from standardized factor loadings, which must be at least 0.5, and its calculation is based on composite reliability (CR) and average variance extracted (AVE) [45].

CR evaluates how well the various items of a scale measure the same conceptual variable, while AVE reflects the total amount of variation in the structure that is due to the variation in the determinants. In our models, many factor loadings fall below 0.50, although for most items, Likert produces higher values. It should be noted that construct validity is satisfactory if the composite reliability values are greater than 0.60, even if the AVE values are less than 0.50 [51,52]. In this sense, the Likert questionnaire design may be considered acceptable and, in any case, outperforms ISRO. The satisfactory convergent validity of the Likert design is also corroborated by the moderate correlation between the two scales (“Contact with doctors and nurses” and “Organization and planning of care”), whereas in ISRO, the lower correlation might indicate that the two scales might in fact not be measuring the same construct. Finally, the fact that correlations in Likert and ISRO designs are both less than 0.85 implies high discriminant validity [52]. Additional evidence in favor of the higher convergent validity of the Likert format comes from the higher Spearman correlations of the two scales with the item measuring overall satisfaction in the Likert questionnaire.

To summarize, our study has found mixed results regarding the overall performance of the two formats. ISRO had a better structural validity, with Likert demonstrating a weaker CFA fit. On the other hand, Likert showed a better convergent validity in terms of higher factor loadings, composite reliability, and average variance extracted, and stronger correlations between scale scores and overall satisfaction with them. It also showed better internal consistency by means of higher Cronbach’s alpha values. Likert and ISRO had, nevertheless, similar divergent validity, since correlations between scale scores were not unacceptably strong. Content validity was assumed to be the same in this study, since Likert-type items were transformed in a way that was meant to preserve the same content. Finally, there were mixed results regarding the sensitivity of the two formats, with no consistent pattern in ceiling effects at an item- or scale-level in favor of one design over the other, and yet, in general, with slightly higher dispersion in the responses derived with Likert. Study findings are outlined in Table 9.

Other studies have also comparatively examined the two types of questionnaire formatting. Höhne and Krebs (2017) [53], in an experiment in a non-health care context involving students, examined the effect of the response scale direction on response behavior. By varying the scale direction, they found response order effects within the Agree/Disagree but not within the item-specific question format, a result that might have been due to the more thorough consideration students showed while completing the item-specific questions that were not similarly framed, as were the Likert items. Höhne and Lenzner (2018) [31] comparatively examined the cognitive processing of Likert and item-specific items in web surveys, using eye-tracking methodology. Their findings suggest that participants comprehended the questions equally, but that item-specific items required a more intensive processing of response categories, resulting in more well-considered quality responses. Höhne, Revilla, and Lenzner (2018) [30] investigated the cognitive effort and response quality associated with Likert and item-specific items in surveys conducted via PCs and smartphones. They applied a split-ballot design defined by device type and question format and found longer response times for item-specific questions, irrespective of the device type and scale length. Moreover, questions expressed in an item response format yielded better response quality, and the authors concluded that there was a more conscientious response on behalf of respondents to item-specific questions.

Hanson (2015) [48] conducted an experiment with face-to-face interviews and found that, regardless of the items’ response options (Likert, ISRO), a substantial minority of respondents may in fact be answering randomly after observing that respondents gave different answers to identical questions administered 20 min apart. Furthermore, item-specific questions had higher test–retest consistency compared with the Likert-scale items, suggesting a more reliable measurement in that non-health care setting. They concluded that agreement-type statements are associated with lower participant engagement and data quality.

Saris et al. (2010) [32] conducted an experimental, representative survey based on randomized between-subjects comparisons with the complementary use of the multitrait–multimethod approach. Results indicated lower data quality stemming from Likert items compared to item-specific ones.

Dykema et al. (2019) [54] used cognitive interviewing and 64 randomized participants, who completed two questionnaires measuring trust in medical researchers. One questionnaire had the items phrased on an agree–disagree response scale, while the other was based on construct-specific questions. They presented various quality indicators, from which it emerged that reliability was higher for the Likert scale, while there were no differences in validity. Some items in the construct-specific format were, however, more affected by recency effects. The study also documented that the construct-specific items facilitate deeper processing. Complementary qualitative analysis revealed several sources of response-processing difficulties that could explain the observed divergence between the two formats. In fact, the construct-specific format did not consistently produce higher data quality when compared to the Likert scale.

The multi-sectoral review by Dykema et al. (2022) [29] recommended the item-specific format over Likert for conducting a survey. They argued that agree–disagree responses are more often associated with acquiescence bias and response effects, whereas their item-specific counterparts yield superior data quality.

Finally, it should be noted that despite the randomization of patients regarding the two response formats, our study was conducted in a single public university hospital. In fact, convenience sampling was employed, and the collection period was rather short (5 weeks). There has never been a representative national survey conducted in this country measuring patient satisfaction. A recent Greek study also based on a convenience sample from hospitals in the Attica region had older, less educated, and more financially constrained patients [55]. In the present study, it may be that the university status of the hospital studied, the proximity of a non-university hospital, the rural areas served, and the varying willingness of individuals with different socio-demographic characteristics to participate have affected sample composition. It follows that the higher educational levels and younger ages might have affected questionnaire completion, potentially facilitating comprehension and use of the ISRO format, which is thought to be more cognitively demanding than the Likert scale.

More generally, since patient satisfaction may be affected by factors such as university hospital status, patient-mix characteristics and expectations, organizational culture, care processes, and workflow, the observed response patterns and overall findings are context-specific and should be treated with caution.

## 5. Conclusions

This study compared a Likert and an item-specific response format for measuring patient satisfaction with hospital care. Some differences were in fact evident in item and scale scores between the two designs, but their magnitude was generally small and unlikely to affect decision-making in most health care contexts. Psychometric testing yielded mixed results. Likert scales had somewhat higher internal consistency and convergent validity, while ISRO questions achieved a better model fit in confirmatory factor analysis. Moreover, no consistent evidence of acquiescence bias was detected. It follows that this form of response bias may be less problematic—at least in a health care setting—than often assumed. Overall, our findings confirm that response format can influence results, but both Likert and ISRO have strengths and limitations. The choice should be guided by study objectives and the intended use of the results.

In particular, the superior model fit of the ISRO format indicates that it may more accurately reflect the underlying structure of patient satisfaction and could therefore be preferred in research that aims to explore or refine conceptual models. Conversely, the higher internal consistency and convergent validity of the Likert format suggest greater reliability and comparability, making it more appropriate for routine monitoring and benchmarking of hospital performance. Thus, the choice between formats should depend on whether the primary goal is theoretical model development (favoring ISRO) or consistent measurement and tracking over time (favoring Likert).

Nevertheless, this study has some limitations. Firstly, it is based on a convenience sample and therefore the results are not generalizable. Although our work aimed to undertake a methodological comparison of response scales and not a measurement of patient satisfaction of the general population of hospitalized patients, it is still true that its external validity is limited to similar public hospital settings and workflows. Secondly, the psychometric performance of both response formats was only partially satisfactory. This is so despite the fact that our initial instrument, using a Likert scale, has, in fact, been previously validated in the Greek language using exploratory factor analysis and other statistical tests and coefficients. In any case, our findings regarding differences between the Likert and ISRO designs should be viewed as indicative rather than conclusive. Thirdly, although care was taken to align the content of the Likert and ISRO versions, subtle differences in how respondents perceived the items may have influenced their answers. Fourthly, this study examined acquiescence bias, but not other sources of measurement error (e.g., social desirability, random responding). Fifthly, the study adopted a between-subjects (randomized) design. While our findings remained largely unchanged across length of stay strata and there were no significant differences in various recorded patient characteristics in this study, the possibility of confounding due to unobserved factors (such as service line and illness acuity) influencing satisfaction scores cannot be precluded. Finally, comparisons with PREMs (patient-reported experience measures) could provide additional insight into how evaluative versus experience-based items perform in this context.

Future research should further assess the two design formats, Likert and ISRO, using different items and questionnaires and larger and more diverse samples. It would also be useful to employ other methods (e.g., cognitive interviews with patients, eye-tracking) to better understand how patients process different response formats. Other forms of error, reliability, and validity (e.g., test–retest reliability, predictive validity) can be assessed with additional methodologies (e.g., multitrait–multimethod, Rasch analysis) to further explore if Likert and ISRO behave differently [56,57]. Finally, the potential influence of different response formats on policy decisions and decision-making processes should be explicitly clarified in light of emerging evidence.

## Figures and Tables

**Table 1:** Participants’ characteristics in the two questionnaires.

	Likert		ISRO		
	n	%	n	%	*p*
Gender					0.418 ^1^
Male	88	44.0%	80	40.0%	
Female	112	56.0%	120	60.0%	
Age (years)					0.416 ^1^
18–30	19	9.5%	22	11.0%	
31–44	33	16.5%	46	23.0%	
45–60	64	32.0%	54	27.0%	
61–74	56	28.0%	56	28.0%	
75+	28	14.0%	22	11.0%	
Level of education					0.791 ^2^
No formal education	2	1.0%	0	0.0%	
Elementary	17	8.5%	18	9.0%	
Secondary	58	29.0%	56	28.0%	
Tertiary	111	55.5%	116	58.0%	
Postgraduate/PhD	12	6.0%	10	5.0%	
Household’s financial condition					0.201 ^2^
Unable to make ends meet	1	0.5%	2	1.0%	
Manage with great difficulty	28	14.0%	40	20.0%	
Manage with little left over	141	70.5%	122	61.0%	
Financially comfortable	30	15.0%	36	18.0%	
Self-reported health status					0.097 ^2^
Very good	56	28.0%	78	39.0%	
Good	117	58.5%	100	50.0%	
Fair	21	10.5%	15	7.5%	
Poor	5	2.5%	7	3.5%	
Type of admission					0.920 ^1^
Planned	88	44%	87	43.5%	
Emergency	112	56%	113	56.5%	
	M	SD	M	SD	*P*
Length of Stay (Days)	5.47	3.68	4.68	2.17	0.040 ^3^

Note: ^1^ χ^2^ test; ^2^ Fisher’s exact test; ^3^ Mann–Whitney test; M: Mean; SD: Standard deviation; *p*: *p*-value.

**Table 2:** Patients’ satisfaction ratings in Likert- and ISRO-formatted questionnaires.

Item	Scale	M	SD	IQR	Md	Min	Μax	CE(%)	*p*
Interest of nursing staff	Likert	4.41	1.00	1.00	5.00	1.00	5.00	66.5	0.381
	ISRO	4.62	0.59	1.00	5.00	3.00	5.00	67.0	
Politeness of nursing staff	Likert	4.62	0.65	1.00	5.00	1.00	5.00	67.5	0.022
	ISRO	4.76	0.48	0.00	5.00	3.00	5.00	77.5	
Clarity of instructions of nursing staff for procedures	Likert	3.55	1.56	3.00	4.00	1.00	5.00	40.0	<0.001
	ISRO	4.76	0.43	0.00	5.00	4.00	5.00	76.0	
Willingness of nursing staff to answer questions	Likert	4.42	0.68	1.00	5.00	2.00	5.00	50.5	0.421
	ISRO	4.49	0.59	1.00	5.00	3.00	5.00	54.0	
Discussion of concerns and fears with nursing staff	Likert	3.77	1.08	2.00	4.00	1.00	5.00	27.5	<0.001
	ISRO	4.39	0.60	1.00	4.00	3.00	5.00	44.5	
Promptness of nursing staff	Likert	4.33	0.95	1.00	5.00	1.00	5.00	57.5	0.003
	ISRO	4.65	0.52	1.00	5.00	3.00	5.00	67.0	
Respect shown by nursing staff	Likert	4.75	0.45	0.00	5.00	3.00	5.00	75.5	<0.001
	ISRO	4.89	0.31	0.00	5.00	4.00	5.00	89.0	
Discretion of nursing staff	Likert	4.81	0.40	0.00	5.00	4.00	5.00	80.5	0.568
	ISRO	4.82	0.43	0.00	5.00	3.00	5.00	83.0	
Waiting time for a bed	Likert	4.03	1.00	1.00	4.00	1.00	5.00	38.0	<0.001
	ISRO	3.34	1.02	1.00	3.00	1.00	5.00	10.0	
Pleasant admission process	Likert	4.03	1.02	1.00	4.00	1.00	5.00	38.0	0.284
	ISRO	4.01	0.83	2.00	4.00	2.00	5.00	31.0	
Well-organized hospital stay without errors	Likert	4.57	0.70	1.00	5.00	1.00	5.00	64.5	0.020
	ISRO	4.50	0.52	1.00	5.00	3.00	5.00	51.0	
Timeliness of scheduled procedures	Likert	3.34	0.96	1.00	4.00	1.00	5.00	7.0	<0.001
	ISRO	3.01	0.76	0.00	3.00	1.00	5.00	4.0	
Discharge before patient feels ready for safe transition	Likert	4.77	0.46	0.00	5.00	3.00	5.00	78.5	0.789
	ISRO	4.79	0.44	0.00	5.00	3.00	5.00	79.5	
Monitoring of patients’ health by doctors	Likert	4.70	0.51	1.00	5.00	2.00	5.00	72.0	<0.001
	ISRO	4.43	0.61	1.00	4.00	3.00	5.00	49.0	
Health-related info provided to patients by doctors	Likert	4.75	0.52	0.00	5.00	2.00	5.00	77.5	0.931
	ISRO	4.76	0.46	0.00	5.00	3.00	5.00	77.0	
Discretion of medical staff	Likert	4.85	0.40	0.00	5.00	3.00	5.00	86.0	0.984
	ISRO	4.85	0.39	0.00	5.00	3.00	5.00	86.0	
Availability of staff to answer questions	Likert	4.75	0.54	0.00	5.00	2.00	5.00	78.0	0.618
	ISRO	4.75	0.45	0.00	5.00	3.00	5.00	75.5	
Overall satisfaction	Likert	4.09	0.78	1.00	4.00	2.00	5.00	30.5	0.011
	ISRO	4.30	0.66	1.00	4.00	3.00	5.00	41.0	

Note: M: mean; SD: standard deviation; Md: median; IQR: interquartile range; Min: minimum value; Max: maximum value; CE: ceiling effect (percent scoring at the scale maximum, 5); *p*: *p*-value of the Mann–Whitney *U* test.

**Table 3:** Percentage of respondents answering the top two response categories in Likert- and ISRO-formatted questionnaires.

Item	Likert		ISRO		*p*
	n	%	n	%	
Interest of nursing staff	169	84.5%	189	94.5%	0.001
Politeness of nursing staff	192	96.0%	196	98.0%	0.241
Clarity of instructions of nursing staff for procedures	130	65.0%	200	100.0%	<0.001
Willingness of nursing staff to answer questions	187	93.5%	190	95.0%	0.519
Discussion of concerns and fears with nursing staff	135	67.5%	188	94.0%	<0.001
Promptness of nursing staff	165	82.5%	196	98.0%	<0.001
Respect shown by nursing staff	199	99.5%	200	100.0%	n/a
Discretion of nursing staff	200	100.0%	197	98.5%	n/a
Waiting time for a bed	155	77.5%	98	49.0%	<0.001
Pleasant admission process	158	79.0%	148	74.0%	0.238
Well-organized hospital stay without errors	191	95.5%	198	99.0%	0.032
Timeliness of scheduled procedures	102	51.0%	40	20.0%	<0.001
Discharge before patient feels ready for safe transition	197	98,5%	198	99,0%	0.653
Monitoring of patients’ health by doctors	197	98.5%	188	94.0%	0.018
Health-related info provided to patients by doctors	196	98.0%	197	98.5%	n/a
Discretion of medical staff	197	98.5%	198	99.0%	n/a
Availability of staff to answer questions	196	98.0%	199	99.5%	n/a
Overall satisfaction	166	83.0%	178	89.0%	0.084

Note: The percentages in the Likert format indicate responses that imply a positive rating of a hospital service aspect (i.e., “agree/strongly agree” in positively worded items and “disagree/strongly disagree” in negatively worded items). In the ISRO design, the percentages denote those who answered one of the two categories that imply satisfaction with the hospital service.

**Table 4:** Descriptive statistics for scale scores in Likert- and ISRO-formatted questionnaires.

	Contact with Doctors and Nurses		Organization and Planning of Care	
	Likert	ISRO	Likert	ISRO
M	4.47	4.68	4.15	3.93
SD	0.39	0.23	0.56	0.43
Md	4.58	4.75	4.20	4.00
IQR	0.54	0.25	0.80	0.60
Min	3.25	3.92	2.00	2.60
Max	5.00	5.00	5.00	4.80
CE (%)	6.0	6.5	1.5	0.0
*p*	<0.001		<0.001	

Note: M: mean; SD: standard deviation; Md: median; IQR: interquartile range; Min: minimum value; Max: maximum value; CE (%): ceiling effect (percent scoring at the scale maximum, 5); *p*: *p*-value of the Mann–Whitney *U* test.

**Table 5:** Regression analysis of scale scores on type of questionnaire and length of stay.

Independent Variables	B	Std. Error	Beta	T	*p*	Lower 95% CI	Upper 95% CI
Dependent variable: Contact with doctors and nurses							
Constant	4.731	0.033	n/a	142.052	<0.001	4.665	4.796
Likert/ISRO	−0.197	0.032	−0.295	−6.163	<0.001	−0.260	−0.134
Length of stay	−0.011	0.005	−0.101	−2.102	0.036	−0.021	−0.001
Dependent variable: Organization and planning of care							
Constant	4.006	0.052	n/a	76.323	<0.001	3.903	4.110
Likert/ISRO	0.231	0.050	0.226	4.587	<0.001	0.132	0.330
Length of stay	−0.017	0.008	−0.100	−2.022	0.044	−0.033	0.000

Note: B: unstandardized coefficients; Std. Error: standard errors of estimated coefficients; Beta: standardized coefficients; CI: confidence intervals; Likert/ISRO: dummy variable (Likert = 1, ISRO = 0).

**Table 6:** Regression analysis of scale scores for stratified samples of patients by length of stay.

Independent Variables	All		≤2 Days		≤3 Days		≤5 Days		>5 Days		>6 Days		>7 Days	
	B	*p*	B	*p*	B	*p*	B	*p*	B	*p*	B	*p*	B	*p*
Dependent variable: Contact with doctors and nurses														
Constant	4.731	0.000	4.976	0.000	4.876	0.000	4.899	0.000	4.585	0.000	4.576	0.000	4.575	0.000
Likert/ISRO	−0.197	0.000	−0.160	0.033	−0.156	0.001	−0.207	0.000	−0.178	0.004	−0.196	0.005	−0.204	0.016
LoS (Days)	−0.011	0.036	−0.104	0.577	−0.053	0.234	−0.055	0.001	0.004	0.670	0.005	0.554	0.006	0.561
Dependent variable: Organization and planning of care														
Constant	4.006	0.000	4.185	0.000	4.267	0.000	4.249	0.000	3.740	0.000	3.663	0.000	3.547	0.000
Likert/ISRO	0.231	0.000	0.350	0.001	0.299	0.000	0.221	0.000	0.251	0.013	0.161	0.217	0.290	0.059
LoS (Days)	−0.017	0.044	−0.060	0.807	−0.090	0.161	−0.078	0.002	0.010	0.483	0.020	0.235	0.022	0.237

Note: B: unstandardized coefficients; *p*: *p*-values; Likert/ISRO: dummy variable (Likert = 1, ISRO = 0); LoS: length of stay.

**Table 7:** Goodness-of-fit indices of confirmatory factor analysis.

Index	Acceptable Values	Likert	ISRO
χ^2^	n/a	489.901	238.504
df	n/a	118	120
χ^2^/df	≤3	4.152	1.988
*p*	>0.05	<0.001	<0.001
RMSEA	≤0.08	0.126	0.071
GFI	≥0.9	0.747	0.869
CFI	≥0.9	0.607	0.689
TLI	≥0.9	0.547	0.641

**Table 8:** CFA factor loadings, reliability, and validity measures for the two questionnaires.

Scale/ Item	Item Loading	α	R^2^	CR	AVE	Item Loading	α	R^2^	CR	AVE
	Likert					ISRO				
Contact with doctors and nurses		0.701		0.602	0.247		0.671		0.297	0.206
Interest of nursing staff	0.739	0.649	0.551			0.260	0.650	0.195		
Politeness of nursing staff	0.377	0.660	0.341			0.254	0.635	0.287		
Clarity of instructions of nursing staff for procedures	0.630	0.723	0.163			0.165	0.648	0.149		
Willingness of nursing staff to answer questions	0.256	0.685	0.145			0.422	0.625	0.508		
Discussion of concerns and fears with nursing staff	0.431	0.671	0.161			0.446	0.608	0.559		
Promptness of nursing staff	0.671	0.643	0.506			0.120	0.667	0.054		
Respect shown by nursing staff	0.128	0.691	0.082			0.057	0.669	0.033		
Discretion of nursing staff	0.103	0.692	0.068			0.136	0.653	0.102		
Monitoring of patients’ health by doctors	0.153	0.687	0.090			0.118	0.678	0.038		
Health-related info provided to patients by doctors	0.138	0.692	0.070			0.085	0.656	0.033		
Discretion of medical staff	0.129	0.690	0.103			0.091	0.653	0.056		
Availability of staff to answer questions	0.154	0.693	0.082			0.039	0.670	0.008		
Organization and planning of care		0.677		0.647	0.469		0.497		0.383	0.274
Waiting time for a bed	0.787	0.493	0.627			0.605	0.358	0.351		
Pleasant admission process	0.912	0.497	0.805			0.558	0.310	0.451		
Well-organized hospital stay without errors	0.180	0.705	0.066			0.127	0.495	0.060		
Timeliness of scheduled procedures	0.451	0.659	0.223			0.234	0.470	0.095		
Discharge before patient feels ready for safe transition	0.126	0.685	0.076			0.098	0.500	0.051		

Note: α: Cronbach’s alpha coefficient for the scale and for the scale if item deleted; R^2^: squared multiple correlation; CR = composite reliability; AVE: average variance extracted.

**Table 9:** Comparative summary of instrument performance: Likert vs. ISRO.

Criterion	Findings Summary	Interpretation	Better Format
Structural validity	ISRO showed better model fit in CFA (RMSEA = 0.071 vs. 0.126); CFI/TLI higher	Indicates more accurate representation of latent structure	ISRO
Convergent validity	Likert showed higher factor loadings, Composite Reliability (CR), Average Variance Extracted (AVE)	Suggest stronger inter-item association	Likert
Divergent validity	Moderate (<0.85) correlations between the two satisfaction scales for Likert and ISRO formats	The two scales/constructs were sufficiently distinct and did not excessively overlap	Mixed/ Comparable
Internal consistency reliability	Likert demonstrated higher Cronbach’s alpha values and improved reliability when low-performing items were removed	Construct measured more coherently since items more internally consistent	Likert
Sensitivity	No consistent differences in ceiling effects or response dispersion: Likert slightly higher variability; ISRO slightly higher central tendency	Both instruments capture a similar range of satisfaction responses	Mixed/ Comparable

## Data Availability

The raw data are available from the authors upon request. The data are not publicly available due to privacy and ethical restrictions.

## Abbreviations

The following abbreviations are used in this manuscript:

AVE	Average Variance Explained
CE	Ceiling Effect
CFI	Comparative Fit Index
CFA	Confirmatory Factor Analysis
GFI	Goodness-of-Fit Index
CR	Composite Reliability
df	Degrees of Freedom
ISRO	Item-Specific Response Options
RMSEA	Root Mean Square Error of Approximation
TLI	Tucker–Lewis Index
